# Occupational, Lifestyle, and Medical Risk Factors for Non-Hodgkin Lymphoma: A Case–Control Study in Ethiopia

**DOI:** 10.3390/cancers17233745

**Published:** 2025-11-24

**Authors:** Obsie T. Baissa, Fozia Abdela, Fissehatsion Tadesse, Amanuel Damie, Moti Sori, Workagegnehu Hailu, Segenet Bizuneh, Bewketu Abebe, Begashaw Adamu, Gail Amir, Ora Paltiel

**Affiliations:** 1 Braun School of Public Health and Community Medicine, Hadassah Medical Organization, Faculty of Medicine, Hebrew University of Jerusalem, Jerusalem 91120, Israelorap@hadassah.org.il (O.P.); 2 Department of Internal Medicine, College of Health Sciences, Addis Ababa University, Addis Ababa P.O. Box 1176, Ethiopia; fozabdela@gmail.com (F.A.); fishtadat@yahoo.com (F.T.); 3 Department of Pathology, College of Health Sciences, Addis Ababa University, Addis Ababa P.O. Box 1176, Ethiopia; amanuel.damie@aau.edu.et (A.D.); motisori@yahoo.com (M.S.); 4 Department of Internal Medicine, College of Medicine and Health Sciences, University of Gondar, Gondar P.O. Box 196, Ethiopia; workhailu@yahoo.com (W.H.); segenet26@gmail.com (S.B.); 5 Department of Pathology, College of Medicine and Health Sciences, University of Gondar, Gondar P.O. Box 196, Ethiopia; 6 Department of Pathology, Hadassah Medical Organization, Faculty of Medicine, Hebrew University of Jerusalem, Jerusalem 91120, Israel; 7 Department of Hematology, Hadassah Medical Organization, Faculty of Medicine, Hebrew University of Jerusalem, Jerusalem 91120, Israel

**Keywords:** lymphoma, NHL, risk factors, Sub-Saharan Africa, case-control study, occupational exposures

## Abstract

**Background:** Non-Hodgkin lymphoma (NHL) is a significant public health concern in Ethiopia. However, data on non-infectious risk factors are scarce. **Methods:** We conducted an age- and sex-matched hospital-based case–control study at two referral hospitals in Ethiopia, enrolling 207 NHL cases and 405 controls. Self-reported occupational, medical, and lifestyle-related exposures were collected via structured interviews. Conditional logistic regression was used to estimate adjusted odds ratios (AOR) and 95% Confidence Intervals (CIs), adjusting for education and residence. **Results:** NHL cases were predominantly male (64.3%) with the majority diagnosed between ages 36–65. Low-grade NHL was the most common subtype (56.4%). Rural residence (AOR = 2.62, 95% CI: 1.71–4.0) and lower educational level (OR = 2.08, 95%CI: 1.33–3.15) were significantly associated with NHL. Occupational exposures, including gardening (AOR = 1.68, 95% CI: 1.04–2.2), pesticide use (AOR = 2.14, 95% CI: 1.34–3.40), agricultural work (AOR = 1.76, 95% CI: 1.12–2.83), and keeping farm animals within the household (AOR = 2.41, 95% CI: 1.51–3.84) were linked to higher NHL odds. In contrast, smoking (AOR = 0.31, 95% CI: 0.17–0.55) and alcohol consumption (AOR = 0.55, 95% CI: 0.35–0.87) were inversely associated with NHL. A history of infection requiring hospitalization (AOR = 2.12, 95% CI: 1.15–3.96) and tonsillectomy/uvulectomy (AOR = 2.61, 95% CI: 1.19–5.71) were associated with increased NHL odds. Subgroup analysis revealed stronger associations between occupational exposures and high-grade NHL, while low-grade NHL was linked to tonsillectomy/uvulectomy. **Conclusions:** Occupational, medical, and lifestyle-related factors are associated with NHL risk in Ethiopia.

## Simple Summary

Non-Hodgkin lymphoma is a growing public health concern in Ethiopia, yet evidence on non-infectious risk factors remains limited. In this hospital-based case–control study including 207 confirmed NHL cases and 405 matched controls, we assessed occupational, lifestyle, and medical history factors. Rural residence, employment in agriculture, pesticide exposure, and keeping farm animals near or around home were associated with higher odds of NHL, while no clear associations were observed with fruit consumption or body mass index. History of tonsillectomy and childhood infection-related hospitalization were also linked to increased risk. The results emphasize the importance of environmental and occupational exposures in the etiology of lymphoma in Ethiopia and highlight the need for further studies.

## 1. Introduction

Lymphomas account for over half of hematologic cancers in Sub-Saharan Africa (SSA), with non-Hodgkin lymphoma (NHL) comprising 80% of the cases [1]. The GLOBOCAN 2022 report cites over 34,000 newly reported cases of NHL in SSA and nearly 22,000 deaths, yielding a mortality-to-incidence ratio (MIR) of 0.65—twice that of high-income countries [2]. In Ethiopia, the Addis Ababa Cancer Registry ranks NHL as the second-most common cancer in men and sixth-most common in women, responsible for 10% and 4% of cancers, respectively [3]. However, the actual burden of NHL is likely underestimated due to the limited geographic coverage of registries and diagnostic capacities [4,5].

The InterLymph Consortium, an international collaborative group, has enhanced our understanding of NHL’s etiologic basis through large-scale pooled analyses, drawing primarily from studies conducted in North America, Europe, and Australia [6]. Established risk factors include male sex, immune dysfunction, infections like Epstein–Barr virus and human immunodeficiency virus (HIV), autoimmune diseases, a family history of lymphoproliferative disorders, and exposure to agricultural chemicals [7,8,9,10]. However, populations from SSA are not represented in the consortium. Research from SSA has shown that infectious agents, such as HIV, play a key role in NHL etiology via a mechanism of immune suppression [11]. Data on non-infectious risk factors in SSA remain limited with only a few studies reporting associations with mining and pesticide exposures [12,13]. A South African study linked uranium exposure and mining activities to increased NHL risk, and a Nigerian study reported a higher risk of lymphoid malignancies among agricultural workers exposed to pesticides [12,13]. In Ethiopia, one previous retrospective study linked herbicide exposure and male sex to NHL, though a lack of diagnostic confirmation limits its interpretation [14]. Our study aims to address these gaps by examining self-reported occupational, medical, and lifestyle-related risk factors for NHL in Ethiopia, thereby enriching existing data and identifying any SSA region-specific risk factors.

## 2. Methods

### 2.1. Study Design, Setting and Population

We conducted a hospital-based case–control study at two major referral teaching hospitals in Ethiopia, namely the Addis Ababa University (hereafter Addis) and the University of Gondar Referral Hospital (hereafter Gondar) which, together, serve large catchment areas across the Amhara, Oromia, South West Ethiopia Peoples Region, Sidama Region, and Central Ethiopia people Region. Consecutive, newly diagnosed NHL patients aged 18 years and older seen at hematology clinics and inpatient wards between August 2022 and June 2024 were enrolled. Eligible cases included all newly diagnosed NHL patients confirmed by morphology and immunophenotyping according to the harmonized NCCN guidelines for Sub-Saharan Africa; patients diagnosed by morphology alone were also included [15]. Controls were recruited from hospital visitors or companions of non-hematology patients, excluding blood relatives of individuals with lymphoma or other hematologic malignancies. Controls were individually matched to cases in a 1:2 ratio by sex and age (±5 years). Using hospital visitors as controls helped minimize potential selection bias related to healthcare access, ensuring that both groups represented individuals able to reach tertiary facilities. Data were collected through structured interviews using a questionnaire adapted from the EPILYMPH study and translated into Amharic and Afan-Oromo [16]. The tool captured sociodemographic characteristics, dietary habits, family and medical history, and occupational exposures. Self-reported weight and height prior to illness were recorded, and context-specific items such as history of tonsillectomy or uvulectomy, dietary restrictions, and fasting patterns were included to capture locally relevant exposures.

### 2.2. Study Procedures

Between August 2022 and June 2024, trained nurses approached cases either in the waiting rooms or chemotherapy rooms, explained the study, and administered the questionnaire in a face-to-face interview. The same staff interviewed both cases and controls on the same day.

Pathology reports were retrieved from the electronic medical records. For cases diagnosed by morphology alone with available biopsy blocks, a four-antibody immunohistochemistry (IHC) panel was performed at the Pathology Departments in the Addis and Hadassah hospital (Israel) by senior hematopathologists (AD and GA) to confirm the diagnosis, determine cell lineage and classify tumor grade. Additional stains were used as needed. Diagnostic confirmation followed the NCCN Harmonized Guidelines for Sub-Saharan Africa, which adapt WHO lymphoma classifications for resource-limited settings [15]. Immunophenotyping was performed using a four-antibody panel: CD20 (B-cell lineage marker; mouse monoclonal, clone L26; Cell Marque, Rocklin, CA, USA), CD3 (T-cell lineage marker; rabbit monoclonal, clone MRQ-39; Cell Marque, Rocklin, CA, USA), CD30 (activation and anaplastic marker; mouse monoclonal, clone Ber-H2; Cell Marque, Rocklin, CA, USA), and Ki-67 (proliferation index; rabbit monoclonal, clone SP6; Cell Marque, Rocklin, CA, USA). This panel distinguishes major B- and T-cell categories and grades of proliferation (low vs. high), enabling classification consistent with the WHO 2016 framework. Additional stains were applied when morphology or lineage was uncertain. Full WHO classification was not feasible for all cases due to limited antibody availability and cost constraints; however, where complete panels were available, WHO-aligned diagnoses were recorded.

### 2.3. Sample Size

Using Epi-info available from https://www.cdc.gov/epiinfo/index.html (accessed on 14 September 2021), we estimated the required sample size assuming 5% exposure prevalence among controls, two-sided α = 0.05, a 1:2 case–control ratio and an odds ratio of 2.4. Based on the Kelsey method, 201 cases and 402 would provide 80% power and a 95% Confidence Interval (CI).

### 2.4. Statistical Analysis

Analysis was performed using R© (version 4.3.2.) available at https://www.r-project.org/ [17]. Descriptive statistics summarized sociodemographic and exposure variables. Categorical variables were reported as frequencies and percentages; BMI was summarized with means and SD. Missing data were <3% for all exposures except BMI, which was imputed using multiple imputation with sex as a predictor. Missingness was limited to current weight (*n* = 24) and BMI (*n* = 27), accounting for approximately 5% of observations. Therefore, single regression-based imputation was applied: weight was imputed using sex as a predictor, and BMI was subsequently imputed using sex and imputed weight. Only missing values were replaced, assuming data were missing at random (MAR). Conditional logistic regression was used to estimate the association between NHL and each exposure. Multicollinearity was checked via variance inflation factor and tolerance values. Odds ratios (OR) with 95% CIs were calculated, and Wald tests assessed statistical significance (*p* < 0.05). Crude ORs were calculated first, followed by adjusted ORs (AOR) for residence, education, and socioeconomic status (SES). SES was assessed by a composite variable based on housing, water source, and transport. As SES adjustment did not meaningfully change results; it was excluded.

Residence and education were treated as potential confounders due to their distributional differences between cases and controls. We evaluated multicollinearity using variance inflation factors (VIF) and tolerance statistics. All variables demonstrated acceptable levels of independence, with VIF values ranging from 1.16 to 1.73 and tolerance values above 0.5, indicating no significant multicollinearity. Mild but not significant correlations were observed among residence type, pesticide use, gardening, keeping farm animals within residential premises (VIF ≈ 1.7; tolerance ≈ 0.6). To minimize over-adjustment and inflated standard errors, models including these occupational exposures were adjusted for education only. We conducted sensitivity analyses to assess the stability of our results based on the diagnostic method (morphology-only vs. IHC) and subgroup analysis explored association by NHL grade.

## 3. Results

**Figure 1:**
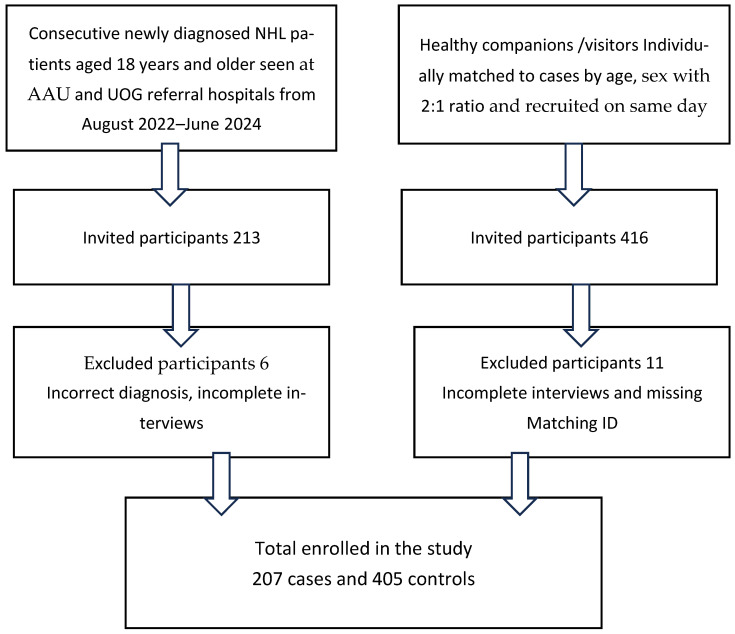
Flow diagram describing the enrollment of NHL cases and controls.

A total of 213 cases were enrolled: six were excluded due to incorrect diagnosis or incomplete interviews, leaving 207 cases for analysis. Figure 1 shows the participant flow diagram. The majority of cases (*n* = 165, 79.6%) were recruited from Addis, the remainder from Gondar. Of the 207 cases, 71.5% (*n* = 148) were diagnosed via morphology and immunophenotyping. Among those without immunophenotyping, 14% *(n* = 29) were cases of chronic lymphocytic leukemia/small-lymphocytic lymphoma (CLL/SLL) diagnosed via bone marrow aspiration and complete blood count, while another 14.5% (*n* = 30) were diagnosed based on morphology alone due to unavailable blocks for further phenotyping. In total, 40.6% (*n* = 81) were high-grade NHL, 56.4% (*n* = 122) low-grade NHL, and four cases were T-cell NHL. Among those with immunophenotyping, CLL/SLL was the most common subtype (36%), followed by DLBCL (35%) and Mantel cell lymphoma (10%). HIV status was recorded for 198 cases. The prevalence was 4.8% (*n* = 10), with eight cases occurring in high-grade NHL and two in low-grade NHL. Thirteen cases (6%) were on tuberculosis treatment during the course of their illness. Controls were not tested for HIV, in accordance with the ethics committee guidance.

Table 1 summarizes sociodemographic and exposure characteristics. Among cases, 64.3% were male. Age distribution was comparable between groups, with 71.5% (*n* = 148) of cases in the 36–50 and 51–65 age groups. Orthodox Christianity was the predominant religion for both cases (68.6%) and controls (72%). Educational level and residence differed by over 10% between cases and controls. Cases were more likely to report employment in agriculture, gardening/backyard farming, keeping farm animals within the household premise, and pesticide use. In contrast, smoking, alcohol use, and regular fruit intake were common among controls. Cases also more frequently reported prior hospitalization for infections and a history of tonsillectomy or uvulectomy.

Table 2 presents the associations between exposures and NHL. Lower educational level (OR = 2.08, 95%CI: 1.33–3.15) and rural residency (OR = 2.62, 95%CI: 1.71–4.0) were associated with increased odds of NHL. Alcohol consumption was inversely associated with NHL (AOR = 0.55, 95%CI: 0.35–0.87) as was smoking history (AOR = 0.31, 95%CI: 0.17–0.55). In contrast, gardening/backyard farming was positively associated with NHL (AOR = 1.68, 95%CI: 1.04–2.2). Fruit intake was not associated with NHL.

Among occupational exposures, agricultural employment was linked to higher NHL odds (AOR = 1.76, 95%CI: 1.12–2.35), as was pesticide exposure (AOR = 2.14, 95%CI: 1.5–3.13) and keeping farm animals within the household premises (AOR = 2.41, 95%CI: 1.51–3.84). Regarding medical history, previous hospitalization for infection (AOR = 2.12, 95% CI: 1.15–3.96) and history of tonsillectomy/uvulectomy (AOR = 2.61,95% CI: 1.19–5.71) were associated with NHL. Increasing BMI was inversely associated with NHL (AOR = 0.88, 95%CI: 0.81–0.95).

### 3.1. Exploratory Subgroup Analysis

Table 3 describes the results of an exploratory subgroup analysis of associations by tumor grade. Occupational exposures showed stronger association withs high-grade NHL. Gardening was significantly associated with high-grade NHL (AOR = 4.08, 95%CI: 1.77–9.4) but not with low-grade NHL. Similarly, pesticide use was linked to high-grade NHL only (AOR = 2.85, 95% CI: 1.2–6.68). Keeping farm animals within residential premises and rural residence were associated with both grades of NHL, with stronger associations for high-grade NHL (AOR = 3.04 and 4.42, respectively).

Alcohol use was associated with lower odds of low-grade NHL (AOR = 0.46, 95%CI: 0.26–0.83), but not with high-grade. Ever-smoking was inversely associated with both low-grade (AOR = 0.40, 95% CI: 0.26–0.89) and high-grade NHL (AOR = 0.18, 95% CI: 0.06–0.55). A history of tonsillectomy/uvulectomy was associated with increased odds of low-grade NHL (AOR = 3.95, 95% CI: 1.23–12.65) but not with high-grade NHL.

### 3.2. Sensitivity Analysis

Appendix A Table A1 shows the sensitivity analysis for 148 cases with immunophenotyping. All lifestyle, occupational, and medical risk factors retained their association and direction in both the crude and adjusted models. No substantial difference (greater than 20%) was noted in effect estimates.

## 4. Discussion

This age- and sex-matched hospital-based case–control study addressed the knowledge gap on risk factors for NHL in Ethiopia, where the lymphoma burden is high but the disease remains under-recognized [4,18]. Over half of the cases were low-grade lymphomas. Most cases were male, primarily aged 36–65. Rural residence, lower education, agricultural work, pesticide use, gardening, and keeping farm animals within residential premises were strongly associated with NHL. In contrast, alcohol consumption and smoking showed inverse associations. Among medical risk factors, prior hospitalization for infection and history of tonsillectomy/uvulectomy were positively associated with NHL. Subgroup analysis showed that gardening, agricultural exposure, and pesticide use were associated only with high-grade NHL. Rural residence and large animal ownership were linked to both tumor grades, with stronger associations for high-grade NHL. Smoking was inversely associated with both tumor grades, while alcohol use and tonsillectomy/uvulectomy were associated only with low-grade NHL.

The sociodemographic profile in our study including age at presentation, male predominance, and low educational level aligns with a previous report from Ethiopia [14]. Low-grade NHL was the most common category. This differs from previous SSA studies and data on Ethiopian-origin Israelis (Personal communication B. Silverman, September 2024) where high-grade NHL dominates [19]. However, our finding aligns with a recent Ethiopian report identifying SLL/CLL as the most frequent NHL, followed by DLBCL [20].

Although HIV prevalence was not assessed among controls, the 4.8% prevalence among cases is nearly five times higher than the national average (0.93%) [21]. Most HIV-positive cases had high-grade NHL consistent with the existing evidence linking HIV to aggressive disease [1]. The overall low HIV prevalence among cases compared to other regions in SSA suggests that HIV may have a limited impact on NHL epidemiology in Ethiopia [1].

Numerous studies have linked pesticide exposure and agricultural work to an increased NHL risk, particularly for high-grade NHL [7,22,23,24]. This is especially relevant in Ethiopia, where 63% of the population is employed in agriculture and 99% of surveyed households report pesticide use often with inadequate safety practices [25,26]. Our findings align with these reports, and with a local Ethiopian study that showed strong associations between NHL and both agricultural work and pesticide use [14]. Gardening was also associated with NHL in our study, mirroring reports from Israel, where rural gardening, specifically growing vegetables and fruits, was linked to increased NHL risk, likely due to chemical exposure [27].

While we observed a positive association between keeping farm animals near or at household premises and NHL, existing evidence is inconsistent. The InterLymph Consortium found no association between livestock farming and NHL, whereas the EPILYMPH and AGRICOHstudies reported increased risk among those working with poultry and beef cattle, suggesting that risk may differ by animal type rather than livestock exposure in general [28,29]. These differences suggest that “farm animal exposure” is not a single uniform exposure. Species-specific environments, tasks, and microbial profiles vary widely and may produce different levels of infectious or immunologic stimulation [29]. The observed association in our study may therefore reflect exposures specific to the types and husbandry practices of animals kept in rural Ethiopian households, rather than a generalized livestock effect.

Among lifestyle-related factors, we found inverse associations between alcohol use and smoking with NHL. Studies from Ethiopia report higher alcohol use in rural areas, consistent with our findings [30,31]. Moreover, the prevalence of current alcohol consumption in our control group (34.1%) aligns with estimates from these studies, confirming that the control groups reflect those of the general population [30,31]. The prevalence of current alcohol consumption in our control group (34.1%) aligns with estimates from population-based studies in Ethiopia. This observed “protective” effect of alcohol aligns with findings from the InterLymph Consortium, particularly for low-grade NHL [32]. The association between smoking and NHL remains inconsistent. Some studies report no association or a modest increase in certain subtypes while others describe nuanced patterns based on intensity, duration, and lymphoma subtype [32,33,34]. In our study, ever-smoking was inversely associated with NHL, but this should be interpreted with caution. Smoking behavior varied by setting: rural cases reported lower smoking rates (8%) compared to urban cases (15%) and rural controls (22%) [35]. Social desirability bias may have led to differential underreporting, by residence area, potentially distorting the observed association. However, the prevalence of ever-smoking among controls (26.9%) is comparable to sub-regional estimates reported in the recent meta-analysis by Daba et al., which documented a smoking prevalence of 24.7% in Oromia and 17.8% in Amhara regions, where the majority of our controls were recruited [35].

We also observed an association between history of hospitalization for infection and NHL risk. This echoes studies from Israel and Sweden that linked early-life infections with NHL, possibly indicating underlying immune dysregulation [27,36,37]. We also noted that history of tonsillectomy/uvulectomy was associated with increased NHL risk. In Ethiopia, these procedures are common and performed traditionally for recurrent throat infections. Traditional uvulectomy is also widely practiced, with one pooled analyses reporting a prevalence of 44% [38]. Studies suggest that tonsillectomy may increase NHL risk due to immune system alterations [39,40]. In the Ethiopian context, such procedures may signal a history of recurrent infections and potential immune dysregulation, contributing to NHL risk.

Our study addresses a critical gap in understanding NHL risk factors in SSA, particularly in Ethiopia, where data on non-HIV-related exposures remain limited. Strengths of this research include its multicenter design and inclusion of both urban and rural population. Immunophenotyping improved diagnostic accuracy and enabled more accurate comparisons. Controls were selected from healthy visitors accompanying non-hematology patients to ensure comparability and reduce healthcare access bias. Although this may have introduced some bias toward younger, urban participants, adjustments for education and residence helped mitigate this effect. Moreover, another strength of our study lies in recognizing that cancer care pathways in low-income countries differ markedly from those in high-income settings, where healthcare access is more uniform. Therefore, residence and educational status, key determinants of patient-related delays and access disparities, were controlled for in the analysis to minimize bias. Our findings remained consistent with previous studies. We also assessed locally relevant exposures, allowing us to explore context-specific factors such as uvulectomy.

However, our study has several limitations. As a case–control study, it is inherently prone to recall bias, particularly for self-reported exposures potentially leading to misclassification of exposures. Limited access to cancer care may have introduced selection bias favoring those who could navigate the health system. Additionally, we could not analyze important risk factors like autoimmune diseases and family cancer history due to low health literacy. Quantification of exposures (e.g., pesticide use, alcohol, smoking) was not feasible, limiting dose–response analysis. Residual confounding from unmeasured factors (e.g., detailed occupational histories) and limited power for rare exposures or subtype-specific analyses are further limitations. We did not quantify response rates; however, the overall response rate among cases appeared to be very high based on assessments using hospital registries. Similarly, based on interviews with data collectors, refusal rates among controls were reported to be minimal.

## 5. Conclusions

Our study provided important evidence linking occupational, lifestyle, and medical risk factors associated with NHL in Ethiopia. The strong associations observed for agricultural work, pesticide use, and animal exposure underscore the role of environmental determinants in lymphoma etiology in Ethiopia. These findings emphasize the need for context-specific research to better understand NHL etiology in the SSA region where subsistence farming is central to the livelihoods of majority of the population, and exposure to factors such as pesticides are a daily reality.

## Figures and Tables

**Table 1:** Distribution of sociodemographic, occupational, lifestyle, and medical exposure variables among non-Hodgkin lymphoma cases and sex and age-matched controls.

Sociodemographic Characteristics of NHL Cases and Age- and Sex-Matched Controls					
Variables		Cases (*n* = 207)	% Cases	Controls (*n* = 405)	% Controls
Study site	Addis Ababa	165	79.6	329	81.2
	Gondar	42	20.3	76	18.8
Sex	Male	133	64.3	275	67.9
	Female	74	35.7	128	31.6
Age (years)	18–35	27	13	70	17.4
	36–50	71	34.3	135	33.6
	51–65	77	37.2	155	38.6
	≥66	32	15.5	41	10.2
Marital status	Married	141	68.1	289	71.4
	Divorced	10	4.8	17	4.2
	Widowed	17	8.2	19	4.7
	Single	38	18.4	77	19
Educational status	≤Primary	140	67.6	223	55.5
	≥High school	66	31.9	177	44
Religion	Orthodox Christian	142	68.6	289	72
	Muslim	32	15.5	65	16.2
	Protestant	31	15	43	10.8
	Others	2	1	4	1
Birth order	First-born	61	29.5	129	32.1
	Second-born	63	30.4	104	25.9
	Third-born or later	81	39.1	165	41.1
Residence	Urban	110	53.1	283	70.9
	Rural	96	46.4	116	29.1
Lifestyle and Occupational Exposures among NHL Cases and Controls					
Variables		Cases (*n* = 207)	% Cases	Controls (*n* = 405)	% Controls
Smoking status	Never smoker	175	84.5	288	73.1
	Former/current smoker	24	11.6	106	26.9
Alcohol consumption	Yes	53	25.6	137	34.1
Fruit intake	≤Once/week	128	61.8	227	57.6
	>Once/week	75	36.2	167	42.4
History of agricultural employment	Yes	65	31.4	80	19.8
Gardening/backyard farming	Yes	58	28	68	16.9
Self-reported pesticide use	Yes	67	31.9	74	18.4
Presence of farm animals in home premise	Yes	66	31.9	71	17.7
Self-Reported Medical History among NHL Cases and Controls					
Variables		Cases *(n* = 207)	% Cases	Controls *(n* = 405)	% Controls
Overweight (BMI ≥ 25 g/m^2^)	Yes	14	6.7%	52	12.9%
History of hospitalization for infection during childhood	Yes	33	15.9	46	11.4
History of tonsillectomy/uvulectomy	Yes	16	8.2	14	3.5
Diabetes/Prediabetes	Yes	5	2.4	14	3.5
History of dental extraction(s)	Yes	83	40.1	182	45.3
	No	112	54.1	210	53.4

Values are presented as frequencies and percentages. BMI = Body Mass Index; SD = Standard Deviation; OR = Odds Ratio; CI = Confidence Interval. Residence classified as urban or rural based on administrative definitions.

**Table 2:** Association between sociodemographic, lifestyle, occupational, and medical factors and risk of non-Hodgkin lymphoma.

Exposures		Crude OR (95 % CI)	OR (Adjusted) **
Education	Uneducated (ref: Educated)	2.08 (1.33–3.15)	
Residence	Rural (ref: Urban)	2.62 (1.71–4.00)	
Birth order	First-born (ref: Fourth or later)	1.11 (0.69–2.43)	1.14 (0.66–1.91)
	Second-born	1.24 (0.72–2.14)	1.25 (0.69–2.11)
	Third-born	1.65 (0.92–2.95)	1.58 (0.82–2.75)
Alcohol consumption	Yes (ref: No)	0.53 (0.34–0.84)	0.55 (0.35–0.87)
Smoking status	Ever smoker (ref: Never smoker)	0.29 (0.17–0.51)	0.31 (0.17–0.55)
Fruit intake >once per week	Yes (ref: No)	0.74 (0.48–1.15)	0.86 (0.55–1.35)
Gardening/backyard farming	Yes (ref: No)	2.01 (1.31–3.11)	1.68 (1.04–2.20)
Agricultural employment ***	Yes (ref: No)	2.15 (1.38–3.34)	1.76 (1.12–2.83)
Pesticide use ***	Yes (ref: No)	2.43 (1.56–3.81)	2.14 (1.34–3.40)
Presence of farm animals within the household premises ***	Yes (ref: No)	2.77 (1.76–4.36)	2.41 (1.51–3.84)
Previous hospitalization for infection during childhood	Yes (ref: No)	1.55 (1.30–1.97)	2.12 (1.15–3.96)
Tonsillectomy/uvulectomy	Yes (ref: No)	2.61 (1.18–5.20)	2.61 (1.19–5.71)
BMI (Kg/m^2^)	Continuous	0.86 (0.80–0.92)	0.88 (0.81–0.95)

OR = Odds Ratio, CI = Confidence Interval, ref = reference category. ** Adjusted for education level and urban/rural residence. *** Due to concerns of over-adjustment, these exposures were adjusted only for education as they are related to (and collinear with) rural/residence.

**Table 3:** Exploratory subgroup analysis of the association between demographic, lifestyle, occupational and self-reported medical exposures, and low- and high-grade lymphomas.

		High-Grade NHL (*n* = 81)		Low-Grade NHL (*n* = 123)	
Exposures	Category (Reference)	Crude OR (95 % CI)	OR (Adjusted) **	Crude OR (95% CI)	Adjusted OR (95% CI)
Education	Uneducated (ref: Educated)	2.27 (1.02–4.76)	--	2.26 (1.33–4.16)	--
Residence	Rural (ref: Urban)	4.42 (1.95–8.95)	--	2.04 (1.20–3.47)	--
Birth order	First-born (ref: ≥Fourth-born)	1.42 (0.65–3.09)	1.22 (0.55–2.78)	1.37 (0.74–2.54)	1.13 (0.60–2.13)
	Second-born	1.08 (0.54–1.91)	1.03 (0.68–2.45)	1.33 (0.97–2.65)	1.7 (0.89–2.54)
	Third-born	1.1 (0.69–1.87)	1.17 (0.75–2.01)	1.29 (0.85–2.01)	1.23 (0.95–2.16)
Alcohol consumption	Yes (ref: No)	0.70 (0.33–1.52)	0.70 (0.31–1.62)	0.45 (0.25–0.79)	0.46 (0.26–0.83)
Smoking status	Ever smoker (ref: Never smoker)	0.16 (0.05–0.47)	0.18 (0.06–0.55)	0.40 (0.21–0.78)	0.40 (0.26–0.89)
Fruit intake >once per week	Yes (ref: No)	0.86 (0.41–1.76)	0.86 (0.41–1.76)	0.65 (0.38–1.12)	0.71 (0.40–1.25)
Gardening/backyard farming	Yes (ref: No)	4.47 (1.96–10.20)	4.08 (1.77–9.40)	1.49 (0.87–2.54)	1.19 (0.68–2.09)
Agricultural employment ***	Yes (ref: No)	2.57 (1.20–5.52)	2.19 (0.99–4.80)	2.04 (1.17–3.56)	1.60 (0.89–2.88)
Pesticide use ***	Yes (ref: No)	3.56 (1.60–7.92)	2.85 (1.20–6.68)	2.10 (1.21–3.64)	1.74 (0.99–3.11)
Presence of farm animals within the household premises ***	Yes (ref: No)	3.32 (1.55–7.10)	3.04 (1.41–6.36)	2.40 (1.36–4.25)	1.91 (1.04–3.49)
Tonsillectomy/uvulectomy	Yes (ref: No)	1.59 (0.54–4.66)	1.34 (0.44–4.06)	3.45 (1.15–10.31)	3.95 (1.23–12.65)
BMI (Kg/m^2^)	Continuous	0.85 (0.76–0.95)	0.87 (0.77–0.98)	0.87 (0.78–0.95)	0.89 (0.81–0.99)

OR = Odds Ratio, CI = Confidence Interval, ref = reference category. ** Adjusted for education level and urban/rural residence. *** Due to concerns of over-adjustment, these exposures were adjusted only for education as they are related to (and collinear with) rural/residence.

## Data Availability

Datasets generated and analyzed during the current study are available from the corresponding author upon reasonable request and with appropriate institutional approvals.

## Appendix A

**Table A1:** Association between lifestyle and occupational and self-reported medical exposures and non-Hodgkin lymphoma restricted to cases with immunophenotyping—conditional logistic regression *.

Exposures	Category	Crude OR (95% CI)	Adjusted ** OR (95% CI)
Education	Uneducated (ref: Educated)	2.08 (1.21–3.77)	-
Residence	Rural (ref: Urban)	1.95 (1.19–3.21)	-
Alcohol consumption	Yes (ref: No)	0.52 (0.30–0.90)	0.55 (0.32–0.96)
Smoking status	Ever smoker (ref: Never smoker)	0.27 (0.14–0.54)	0.30 (0.15–0.60)
Fruit intake >once per week	Yes (ref: No)	0.80 (0.48–1.33)	0.96 (0.57–1.62)
Gardening/backyard farming ***	Yes (ref: No)	1.95 (1.17–3.28)	1.68 (0.99–2.88)
Agriculture employment ***	Yes (ref: No)	1.99 (1.19–3.32)	1.73 (1.01–2.93)
Pesticide use ***	Yes (ref: No)	2.50 (1.45–4.35)	1.99 (1.1–3.56)
Presence of farm animals within the household premises	Yes (ref: No)	2.67 (1.58–4.5)	2.07 (1.15–3.72)
Birth order	First-born (ref: ≥Fourth-born)	1.27 (0.73–2.20)	1.07 (0.62–1.88)
BMI	Continuous	0.88 (0.81–0.96)	0.90 (0.82–0.99)
Tonsillectomy/uvulectomy	Yes (ref: No)	3.03 (1.17–7.85)	3.0 (1.11–8.1)

* Cases and controls matched for age and sex. ** Adjusted for education and urban/rural residence. *** For concerns of over- adjustment, these exposures were adjusted only for education as they are related to (and collinear with) rural/residence. OR = Odds Ratio, CI = Confidence Interval, Ref = Reference Category.
